# The mediating role of pregnancy-related anxiety between fetal movement self-monitoring adherence and maternal-fetal attachment in the third trimester

**DOI:** 10.3389/fgwh.2026.1710272

**Published:** 2026-04-08

**Authors:** Xingshan Zheng, Ruixia Wu, Lishan Lin, Wenyu Huang, Nengqing Liu, Jiaqi Wu

**Affiliations:** 1 Department of Obstetrics, Boai Hospital of Zhongshan, Zhongshan, China; 2 Department of General Affairs, Boai Hospital of Zhongshan, Zhongshan, China; 3 Department of Emergency, Boai Hospital of Zhongshan, Zhongshan, China; 4 Reproductive Medicine Center, Boai Hospital of Zhongshan, Zhongshan, China

**Keywords:** fetal movement self-monitoring, maternal-fetal attachment, mediating effect, pregnancy-related anxiety, third trimester

## Abstract

**Background:**

This study aimed to investigate the mediating role of pregnancy-related anxiety (PRA) in the relationship between fetal movement self-monitoring adherence (FMSMA) and maternal–fetal attachment (MFA) among women in the third trimester.

**Methods:**

A cross-sectional study was conducted among 348 pregnant women in their third trimester recruited from the Boai Hospital of Zhongshan between January and December 2024. Data were collected using a general information questionnaire, the FMSMA Scale (FMSMAS), the Pregnancy-Related Anxiety Scale, and the Maternal Antenatal Attachment Scale. Pearson's correlation analysis was used to examine the relationships among the variables, and the mediating effect was tested using Hayes' PROCESS macro (Model 4).

**Results:**

FMSMA was positively correlated with MFA (*r* = 0.291, *p* < 0.001) and negatively correlated with PRA (*r* = –0.221, *p* < 0.001). PRA negatively correlated with MFA (*r* = –0.182, *p* < 0.001). After controlling for sociodemographic and obstetric characteristics, the mediation analysis indicated that FMSMA had a significant indirect effect on MFA through reduced PRA (indirect effect = 0.088, Bootstrap 95% CI: 0.043–0.133 M, *p* < 0.05), accounting for 30.66% of the total effect.

**Conclusion:**

PRA partially mediates the relationship between FMSMA and MFA. Enhancing adherence to self-monitoring may help reduce pregnancy-related anxiety and strengthen maternal–fetal attachment. However, due to the cross-sectional nature of this study, causal relationships cannot be confirmed, and these findings should be interpreted with caution.

## 1 Introduction

Maternal-fetal attachment (MFA) refers to the unique emotional bond formed between a mother and her fetus during pregnancy (1). As a significant predictor of maternal and infant mental health, MFA quality has profound implications for the long-term development of both mothers and children (2). Studies indicate that higher levels of MFA are associated with better prenatal health behaviors (such as attending regular prenatal checkups and maintaining a healthy diet) and contribute to improved postpartum mother-infant emotional bonding, which is closely linked to healthy socioemotional development in early childhood (3–5). However, the establishment and development of MFA can be influenced by various interfering factors. In China, pregnant women experience pregnancy-related anxiety (PRA) because of physiological changes, adjustments in social relationships, and concerns about pregnancy outcomes (6, 7). Such anxiety may reduce maternal attention and emotional investment in the fetus, thereby potentially having a negative impact on MFA (8, 9). This underscores the critical role of the maternal psychological state in the formation of MFA.

Recently, woman-centered proactive intervention strategies have gained increasing attention in the field of perinatal medicine (10–13). Fetal movement self-monitoring is a low-cost and noninvasive technique that designed to enhances maternal perception of fetal movements, helps establish communication and connection with the fetus, and facilitates the formation of embodied cognition (14–16). Theoretical perspectives suggest that improving fetal movement self-monitoring adherence (FMSMA), that is, the extent to which pregnant women correctly and regularly follow clinical guidance to monitor fetal movements, may strengthen the perception of emotional connection to the fetus, thereby being positively related to MFA MFA (17, 18). Simultaneously, this positive bonding experience may also be indirectly associated with MFA by potentially mitigating PRA (17, 19). Based on the existing evidence, we hypothesized that there may be a specific pathway linking FMSMA, pregnancy-related anxiety, and maternal-fetal attachment, in which PRA serves as a mediator in the relationship between monitoring adherence and MFA. Although previous studies have examined the pairwise associations between these variables, few have systematically investigated the integrated mechanisms, particularly with PRA as a mediating variable (17, 20).

This study employs a cross-sectional survey to focus, for the first time, on the potential pathway of “fetal movement self-monitoring adherence—pregnancy-related anxiety—maternal-fetal attachment”. This study aimed to explore the relationship between FMSMA and MFA, and to examine whether PRA plays a mediating role in this relationship. The findings are expected to provide a theoretical basis for understanding the psychological mechanisms underlying the association between FMSMA and MFA, and offer insights for healthcare providers in developing or optimizing prenatal psychological interventions and building high-quality perinatal care models.

## 2 Methods

### 2.1 Participants

Convenience sampling was employed to recruit women in the third trimester of pregnancy who attended regular prenatal checkups at the Department of Obstetrics, Zhongshan Boai Hospital between January 2024 and December 2024. The inclusion criteria were as follows: (1) gestational age ≥ 28 weeks; (2) age > 18 years; (3) ability to read, understand, and communicate effectively; and (4) provision of informed consent to participate in the study. The exclusion criteria were as follows: (1) presence of severe obstetric complications or comorbidities; (2) diagnosis of major physical or psychiatric disorders; and (3) confirmed fetal malformations or twin pregnancies.

### 2.2 Measures

#### 2.2.1 Demographic characteristics

We collected the demographic characteristics of the included pregnant women using a self-designed questionnaire that covered the following variables: age, educational level, household registration status (hukou), monthly household income per capita, type of health insurance, only child status, marital status, whether the pregnancy was planned, parity, pregnancy complications, and history of adverse pregnancy outcomes.

#### 2.2.2 Pregnancy-related anxiety scale

Pregnancy-related anxiety was assessed using the pregnancy-related anxiety scale (PAS) developed by Xiao et al. (21). The validity of this scale has been confirmed in a Chinese female population (21), and its validity has been confirmed, with a Cronbach's *α* coefficient of 0.840. The PAS consists of 13 items grouped into three dimensions: 5 items related to worries about fetal health (Worry Fetal Health), 2 items concerning fear of childbirth (Worry Childbirth), and 6 items addressing self-concern. Responses were recorded on a 4-point Likert scale. The total scores range from 13 to 52, with higher scores indicating more severe pregnancy-related anxiety. A total score of ≥24 was used as the cut-off value to indicate the presence of pregnancy-related anxiety. In the current study, the Cronbach's *α* coefficient for the PAS was 0.869.

#### 2.2.3 Maternal antenatal attachment scale

MFA was assessed using the Maternal Antenatal Attachment Scale (MAAS), originally developed by Condon et al. (22). The Chinese version of the scale was translated and adapted by Nie et al. (23), and its validity has been confirmed in a Chinese female population, with a reported Cronbach's α of 0.77. The MAAS consists of 19 items grouped into two subscales: 11 items evaluating attachment quality and 8 items measuring attachment intensity. Responses were scored on a 5-point Likert scale. The total score ranges from 19 to 95, with higher scores indicating a stronger level of maternal-fetal attachment. In the current study, the Cronbach's α coefficient for the MAAS was 0.751.

#### 2.2.4 Fetal movement self-monitoring adherence scale

The FMSMA Scale (FMSMAS) developed by Guo et al. (24) was used to assess the pregnant women's FMSMA scores. The scale has been validated in a Chinese female population, with a Cronbach's α coefficient of 0.936. It consists of 14 items divided into two dimensions: 10 items pertaining to regular monitoring (Regular Monitoring) and 4 items related to the response to abnormal fetal movements (Vigilance for Anomalies). A dichotomous scoring system is used for each item. The total score ranges from 0 to 14, with higher scores indicating better FMSMA. In the current study, the Cronbach's α coefficient for the FMSMAS was 0.811.

### 2.3 Data collection

Trained researchers screened the electronic medical records of women attending the antenatal clinic to identify potential participants who met the inclusion criteria. Then, the researchers approached the eligible women at the end of their antenatal care visits and explained the study purpose, significance, and confidentiality to the participants face-to-face. Written informed consent was obtained from all participants prior to data collection, and a copy of the signed consent form was offered to them. After informed consent was obtained, an electronic questionnaire was distributed using QR codes. All questionnaires were completed anonymously on-site by scanning the code or clicking the provided link, and were submitted immediately upon completion. According to Kendall's sample size estimation method (19), the required sample size should be 5–10 times the number of items in the questionnaire. Taking the Maternal Antenatal Attachment Scale (MAAS), which has the most items (19) as a reference and accounts for a 20% non-response rate, the minimum sample size was calculated to be between 114 and 228. A total of 375 questionnaires were distributed and 348 were returned, yielding a valid response rate of 92.8%.

### 2.4 Ethics statement

This study was approved by the Medical Ethics Committee of Zhongshan Boai Hospital (Approval No. KY-2024-004-61). The title and introduction of the online questionnaire provided details regarding the nature and purpose of the research, and information about the research team. Participants were assured that their involvement was voluntary and informed consent was obtained. The questionnaire did not collect any personally identifiable information and all responses were anonymized. All data were handled confidentially in accordance with the data protection regulations.

### 2.5 Data analysis

Data analysis was performed using IBM SPSS Statistics version 26.0. Categorical variables were presented as frequencies and percentages, while continuous variables that followed a normal distribution were expressed as mean ± standard deviation (mean ± SD). An independent samples *t*-test was used for comparisons between two groups, and a one-way ANOVA was applied for comparisons among multiple groups in the univariate analysis. Correlations between the FMSMA, PRA, and MFA scores were examined using Pearson's correlation analysis. Mediation analysis was conducted using Model 4 (a simple mediation model) of the PROCESS macro for SPSS developed by Hayes, with the significance of the indirect effects tested using the bootstrap method with 5000 resamples. To control for potential confounding effects, covariates including maternal age, education, and average monthly household income and parity were entered into the mediation model. Statistical significance was defined as a two-tailed *p*-value of <0.05.

## 3 Results

### 3.1 Socio-demographic characteristics of the study participants

A total of 348 pregnant women were included in this study, with a mean age of 29.82 ± 4.86 years. The majority of participants (67.5%) held a junior college or bachelor's degree, and most (66.1%) resided in urban areas. Regarding obstetric characteristics, 65.5% were primiparous, and 64.1% had a planned pregnancy. Additionally, 12.4% of the participants reported pregnancy complications, and 28.2% had a history of adverse pregnancy outcomes. Detailed socio-demographic characteristics are presented in Table 1.

**Table 1:** General information of 348 third-trimester pregnant women.

Characteristics	*N*	Percentage (%)	Characteristics	*N*	Percentage (%)
Age			Self-pay	39	11.21
18–25	39	11.21	Other	13	3.73
26–30	165	47.41	Only Child		
31–35	119	34.20	Yes	69	19.83
>36	25	7.18	No	279	80.17
Education			Marital Status		
Junior high school or below	29	8.33	First marriage	250	71.84
High school/technical secondary school	65	18.68	Remarried	98	28.16
Bachelor's degree/college	235	67.53	Planned Pregnancy		
Graduate degree or above	20	5.46	Yes	223	64.08
Household Registration			No	125	35.92
Urban	230	66.10	Parity		
Rural	118	33.90	1	228	65.52
Average monthly household income			≥2	120	34.48
<3,000 RMB	71	20.40	Pregnancy Complications		
3,000–5,000 RMB	143	41.09	Yes	43	12.36
>5,000 RMB	134	38.51	No	305	87.64
Health Insurance Type			Adverse Obstetrical History		
Employee basic medical insurance	114	32.76	Yes	98	28.16
Urban resident basic medical insurance	76	21.84	No	250	71.84
New Rural Cooperative Medical Scheme	106	30.46			

### 3.2 The scores of PSA, FMSMAS and MASS among women in the third trimester

Table 2 summarizes the PSA, FMSMAS and MASS scores among women in the third trimester. The mean total PRA score was 23.87 ± 5.86, of which 35.63% (*n* = 124) had a total score >24. Regarding the subscales of PRA, the subscale scores were 10.19 ± 3.15 for “Worry Fetal Health”, 4.17 ± 1.63 for “Worry Childbirth”, and 9.49 ± 1.85 for “Self-Concern”. The total score of FMSMAS was 8.72 ± 2.91, with subscale scores of 5.63 ± 2.31 for “Regular Monitoring” and 3.07 ± 0.91 for “Vigilance for Anomalies”. The mean total MFA score was 71.97 ± 7.19, consisting of 45.33 ± 3.88 for “Attachment Quality” and 25.96 ± 5.02 for “Attachment Intensity”.

**Table 2:** Scores of pregnancy-related anxiety, fetal movement self-monitoring health belief, and maternal-fetal attachment in third-trimester pregnant women.

Item	Score (Mean ± SD)
Pregnancy-Related Anxiety (PRA)	23.87 ± 5.86
Worry Fetal Health	10.19 ± 3.15
Worry Childbirth	4.17 ± 1.63
Self-Concern	9.49 ± 1.85
Fetal Movement Self-Monitoring Adherence (FMSMA)	8.72 ± 2.91
Regular Monitoring	5.63 ± 2.31
Vigilance for Anomalies	3.07 ± 0.91
Maternal-Fetal Attachment (MFA)	71.97 ± 7.19
Attachment Quality	45.33 ± 3.88
Attachment Intensity	25.96 ± 5.02

### 3.3 The relationships among PRA, FMSMA, and MFA in third-trimester pregnant women

The Pearson correlation matrix for all study variables is presented in Table 3. Notably, PRA demonstrated significant negative correlations with both FMSMA (*r* = −0.221, *p* < 0.001) and MFA (*r* = −0.182, *p* < 0.001). This indicates that lower anxiety levels were associated with higher adherence and stronger maternal-fetal bonding. Conversely, a significant positive correlation was observed between FMSMA and MFA (*r* = 0.291, *p* < 0.001), suggesting that better adherence to fetal movement monitoring is linked to higher levels of attachment.

**Table 3:** Correlations among pregnancy-related anxiety, fetal movement self-monitoring adherence, and maternal-fetal attachment.

Variables	1	2	3	4	5	6	7	8	9	10
1 Total score of PRA	1.000									
2 Worry Fetal Health	0.832***	1.000								
3 Worry Childbirth	0.896***	0.561***	1.000							
4 Self-Concern	0.564***	0.133*	0.127*	1.000						
5 Total score of FMSMA	−0.221***	−0.258***	−0.172**	−0.094	1.000					
6 Regular Monitoring	−0.252***	−0.279***	−0.166**	−0.038	0.813***	1.000				
7 Vigilance for Anomalies	−0.174**	−0.164**	−0.177**	−0.115*	0.742***	0.224***	1.000			
8 Total score of MFA	−0.182***	−0.166**	−0.275***	−0.086	0.291***	0.255***	0.178**	1.000		
9 Attachment Quality	−0.194***	−0.218***	−0.258***	−0.102*	0.277***	0.264***	0.136*	0.826***	1.000	
10 Attachment Intensity	−0.156**	−0.174**	−0.206***	−0.057	0.298***	0.217***	0.182**	0.894***	0.302***	1.000

PRA, pregnancy-related anxiety; FMSMA, fetal movement self-monitoring adherence; MFA, maternal-fetal attachment.

* *p* < 0.05.

** *p* < 0.01.

*** *p* < 0.001.

### 3.4 Mediation analysis

**Figure 1:**
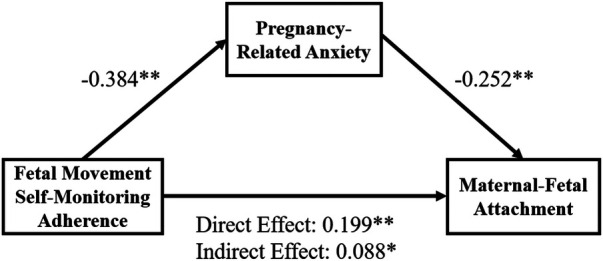
The mediating effect of PRA on the relationship between compliance with FMSMA and MFA in third-trimester women. *p < 0.05, **p < 0.01.

The detailed results of the mediation analysis are presented in Table 4 and Figure 1. In this model, FMSMA, PRA, and MFA were entered as independent, mediator, and dependent variables, respectively. The analysis was conducted using the bootstrap method with 5000 resamples. The results demonstrated a significant total effect of the FMSMA on the MFA (effect = 0.287, *p* < 0.05). Adherence also significantly predicted PRA (effect = −0.384, *p* < 0.05). After controlling for the influence of adherence, PRA remained a significant predictor of MFA (effect = −0.252, *p* < 0.05). The direct effect of adherence to MFA remained significant after accounting for the mediator (effect = 0.199, *p* < 0.05). The indirect effect was statistically significant with a 95% confidence interval that did not include zero. Thus, PRA partially mediated the relationship between FMSMA and MFA, accounting for 30.66% of the total effect (−0.384 × −0.252/0.287 = 0.3066).

**Table 4:** Test of the mediating effect of pregnancy-related anxiety between fetal movement self-monitoring adherence and maternal-fetal attachment .

Item	Effect value	SE	95% CI	Effect size (%)
Total Effect	0.287	0.039	[0.118, 0.280]	100
Direct Effect (X→Y)	0.199	0.041	[0.210, 0.364]	69.34
Indirect Effect (X→M→Y)	0.088	0.023	[0.043, 0.133]	30.66

X, fetal movement self-monitoring adherence; Y, maternal-fetal attachment; M, pregnancy-related anxiety.

## 4 Discussion

Our study examined the levels of PRA, FMSMA, and MFA in women in their third trimester and conducted a mediation analysis among these variables.

In our study, the total PRA score among third-trimester women was a moderate level, which is consistent with findings from other studies in China (7, 25, 26). The incidence of PRA (total score >24) in this study was 35.63%, which is relatively high (international reports range from 10–40%) (7, 27–29). PRA refers to worries or anxieties related to pregnancy concerning oneself, the fetus, and family or social relationships, representing prenatal psychological stress that is distinct from general anxiety during pregnancy (30). PRA is documented to be closely associated with adverse outcomes, such as pregnancy complications, restricted fetal growth, and potential deficits in the child's neurobehavioral development and connection to maternal and infant outcomes may be more significant than general pregnancy anxiety (31–33). Bekkhus et al. (34) found that PRA is linked to an increased risk of spontaneous preterm birth or low birth weight. Shao et al. (29, 35) reported that PRA in the third trimester may affect the altered social and fine motor skill development in infants. Acosta et al. (36, 37) proposed that PRA during pregnancy is related to variations in fetal amygdala development, which may predispose children to emotional and behavioral problems. Kortesluoma et al. (38) further revealed that PRA could increase the risk of mental disorders in offspring through the hypothalamic-pituitary-adrenal axis. The relatively high prevalence of PRA in this study may be related to the trimester, some studies suggest that women in the third trimester were more susceptible to PRA than those in other stages (39, 40). Although we excluded participants with severe obstetric complications, the imminent approach of labor often intensifies specific fears regarding the delivery process and fetal safety, which are core components of PRA. Furthermore, most of our participants were primiparous women. Studies have shown that first-time mothers often experience significantly higher anxiety due to a lack of childbirth experience and uncertainty about their future maternal role (41, 42). Finally, the study setting itself may contribute to the observed anxiety levels. The participants were recruited from a tertiary general hospital, which serves as a regional referral center. Previous studies have shown that women with higher levels of pregnancy-related anxiety or fear of childbirth are more likely to perceive their pregnancy as “risky” and thus prefer giving birth in high-level medical institutions to ensure maximum safety (43). In the Chinese healthcare context, this often manifests as a “siphon effect”, where anxious pregnant women bypass primary care clinics in favor of tertiary hospitals (44). Consequently, our sample likely included a disproportionate number of women with high baseline anxiety. Given the potential adverse effects of PRA on maternal health and long-term child development, proactive clinical prenatal care, with a focus on maternal psychological well-being, is essential to reduce PRA.

The total score of FMSMA among third-trimester women was also at a moderate level, slightly higher than that reported by Guo et al. (24), who investigated 100 third-trimester pregnant women in a tertiary hospital in Inner Mongolia, China, in 2021 and found a medium-low level of adherence (mean score: 7.78 ± 2.23). Numerous studies have shown that abnormal fetal movements are strongly associated with adverse pregnancy outcomes, and the timely identification of warning signs through self-monitoring is an effective way to potentially reduce these risks (45). Moreover, some studies have suggested that regular daily fetal movement self-monitoring may facilitate maternal-infant emotional bonding (17). Therefore, international prenatal care guidelines recommend that women in their third trimester adhere to regular self-monitoring of fetal movements (46, 47). However, FMSMA is generally low among pregnant women, and surveys indicate that only 30%–50% of pregnant women in the third trimester insist on daily self-monitoring (15). In our study, adherence improved compared with previous years, suggesting enhanced awareness and health beliefs, possibly due to socioeconomic development and improved population literacy. Nonetheless, there is a substantial scope for improvement. Healthcare providers should develop effective interventions to enhance awareness and FMSMA, ensure prenatal safety, and promote healthy maternal-fetal emotional development.

The total MFA score among third-trimester women was indicating a moderate to high level, though lower than that reported by Zhang (48), and Zhao (49) in china. The MFA represents a unique emotional bond between mother and fetus. High MFA levels encourage women to actively manage their physical and mental health, prevent prenatal anxiety and postpartum depression, and facilitate the transition to motherhood (1). Conversely, low MFA may hinder health behaviors beneficial to the fetus and adversely affect the formation of future parent-child relationships and the child's development (36). Previous studies have shown that gestational age is a key factor influencing MFA, and as pregnancy progresses and the fetus matures, women's adaptation to pregnancy and perception of the fetus increase, leading to higher MFA levels in the third trimester than in earlier stages (20). Additionally, maternal age and income level were positively correlated with MFA; older women or those with higher incomes may have greater active expectations of the fetus, find the transition to motherhood easier, and invest more emotionally in it. Although all participants in this study were in their third trimester, the higher proportion of younger women and those with low to middle income may explain the lower MFA scores relative to other studies. MFA is a valuable predictor of maternal and infant health, and healthcare providers should continuously monitor MFA levels, identify women with low MFA, and develop targeted, personalized interventions to enhance mother-fetus interactions and improve MFA.

Correlation analysis revealed a positive relationship between FMSMA and MFA, suggesting that better adherence is associated with higher MFA. Mikhail et al. (50) and Esra (18) et al. (13) confirmed through single- and multicenter prospective studies that behavioral interventions involving fetal movement counting significantly improved the MFA, which aligns with our findings. Damato et al. (51) found that mother-fetus communication and maternal perception of fetal movements are crucial factors influencing prenatal MFA. When women count fetal movements, they often interact with the fetus by touching their abdomen, which may increase their sensitivity and emotional connection, and foster a sense of intimacy. However, given the cross-sectional design of this study, the possibility of reverse causality must be considered. It is plausible that women who already possess a stronger emotional attachment to their fetus (high MFA) are more motivated to strictly adhere to fetal movement monitoring guidelines as a protective behavior. Thus, the relationship between adherence and attachment is likely bidirectional. Conversely, adherence to self-monitoring was negatively correlated with PRA. Regular fetal movement counting in the third trimester may help prevent PRA. Some studies have found that daily fetal movement counting promotes relaxation, emotional stability, and better stress management, thereby preventing anxiety and depression (17, 52–54). This may be because counting movements helps women feel more in control and secure about their pregnancy, thereby reducing their PRA. Additionally, PRA negatively correlated with MFA, PRA in the third trimester may lowered MFA levels. Compared to positive information, women tend to focus more on negative aspects, such as concerns about fetal health, fear of childbirth, and self-doubt about motherhood, which collectively contribute to pregnancy-related anxiety (49, 55, 56). PRA can severely affect women's perceptions of pregnancy and the fetus, reduce parenting self-efficacy and emotional investment, hinder effective emotional bonding, and ultimately impair MFA (8, 57, 58).

The mediation analysis indicated that PRA partially mediated the relationship between FMSMA and MFA, accounting for 30.66% of the total effect. This means that adherence not only directly influences MFA, but also indirectly affects it through PRA. Higher adherence involves regular daily fetal movement counting, which serves as a simple yet effective means of mother-fetus communication. This regular interaction may enhance maternal engagement and fetal perception, forming the foundation of the MFA. Furthermore, counting fetal movement is an effective way to assess fetal well-being and ensure safety. Providing real-time feedback on fetal health reduces concerns about unknown risks, promotes psychological relaxation and security, and generates pleasure through interactions, all of which may be alleviate pregnancy-related anxiety. Reduced anxiety frees up emotional resources, allowing women to engage more fully in focused interactions with their fetuses, potentially enhancing MFA. Notably, PRA only partially mediated this relationship, indicating the presence of direct effects independent of the mediator. For instance, the ritualistic nature of fetal movement recordings may directly increase attention to the fetus through operant conditioning, thereby enhancing attachment. Alternatively, the monitoring behavior might be internalized as part of a “responsible mother” social role, directly strengthening attachment motivation through self-consistency theory. In summary, healthcare providers should develop scientific and effective interventions to improve FMSMA, encourage regular monitoring, promote mother-fetus communication and bonding, reduce maternal anxiety, and safeguard prenatal physical and mental health.

Several limitations of this study should be acknowledged. First, the use of convenience sampling from a single tertiary hospital introduces potential selection bias. Women attending regular antenatal care at a tertiary medical center may differ systematically from those in primary care clinics or rural settings. specifically, our participants may possess higher levels of health literacy, better socioeconomic status, and potentially different anxiety levels (e.g., due to high-risk pregnancies often managed in tertiary centers) compared to the general pregnant population. As a result, these differences may influence their adherence behaviors and MFA levels, thereby limiting the external validity (generalizability) of our findings to broader populations. Future studies employing multi-center random sampling, including participants from diverse socio-economic and geographical backgrounds, are recommended to verify these findings. Second, the FMSMA scale used in this study relies on dichotomous scoring, which may limit the variability of responses and reduce sensitivity compared to Likert-type measures. Future studies could consider developing or using continuous measures to capture more nuanced behavioral differences. Third, as the data were collected through self-report questionnaires, the results are subject to self-report measurement errors, such as social desirability bias or recall bias. Fourth, since all variables were measured at a single point in time using the same method, common-method bias cannot be completely ruled out. Finally, the cross-sectional design precludes causal inference. As noted, reverse causality (e.g., high MFA driving better adherence) remains a plausible explanation. Future longitudinal or interventional studies are warranted to elucidate the causal directions among FMSMA, PRA, and MFA.

## 5 Conclusion

In conclusion, this study clarified the relationships between PRA, FMSMA, and MFA in women in their third trimester. This demonstrates that the FMSMA not only directly affects the MFA, but also indirectly influences it through the PRA. These findings suggest that healthcare providers should focus on enhancing the FMSMA and promoting behavioral interventions involving fetal movement counting to alleviate PRA and improve MFA.

## Data Availability

The raw data supporting the conclusions of this article will be made available by the authors, without undue reservation.
